# Prediction of drug hypersensitivity by comprehensive modeling of HLA-peptidomes

**DOI:** 10.1093/bib/bbag350

**Published:** 2026-07-03

**Authors:** Yi Zhong, Volker M Lauschke, Yi Wang, Yitian Zhou

**Affiliations:** Department of Physiology and Pharmacology and Center for Molecular Medicine, Karolinska Institutet and University Hospital, Solnavägen 9, Biomedicum, 17165 Stockholm, Sweden; Pharmaceutical Informatics Institute, College of Pharmaceutical Sciences, Zhejiang University, 866 Yuhangtang Rd, 310058 Hangzhou, China; Department of Physiology and Pharmacology and Center for Molecular Medicine, Karolinska Institutet and University Hospital, Solnavägen 9, Biomedicum, 17165 Stockholm, Sweden; Dr. Margarete Fischer-Bosch Institute of Clinical Pharmacology, Auerbachstr. 11270376 Stuttgart, Germany; University of Tübingen, Keplerstraße 2, 72074 Tübingen, Germany; Department of Pharmacy, the Second Xiangya Hospital, Central South University, No. 139 Renmin Middle Road, Furong District, 410011 Changsha, China; Pharmaceutical Informatics Institute, College of Pharmaceutical Sciences, Zhejiang University, 866 Yuhangtang Rd, 310058 Hangzhou, China; State Key Laboratory of Chinese Medicine Modernization, Innovation Center of Yangtze River Delta, Zhejiang University, Xiangfudang Sci-tech Innovation Green Valley, Jiashan County, 314102 Jiaxing, China; Department of Physiology and Pharmacology and Center for Molecular Medicine, Karolinska Institutet and University Hospital, Solnavägen 9, Biomedicum, 17165 Stockholm, Sweden; Department of Biomedical and Clinical Sciences, Linköping University, Lasarettsgatan 68, Build 420, 58225 Linköping, Sweden; Science for Life Laboratory, Linköping University, Lasarettsgatan 20, Building 463, 58183 Linköping, Sweden

**Keywords:** human leukocyte antigen, drug hypersensitivity, genetic variants, computational modeling, pharmacogenetics

## Abstract

Human leukocyte antigen (HLA)-B*57:01 associated with abacavir-induced hypersensitivity syndrome (ABC-HSS) is one of the most extensively studied immune-mediated drug hypersensitivity reactions (DHRs). The high odds ratio and strong predictive values of HLA-B*57:01 for ABC-HSS have prompted the Food and Drug Administration and European Medicines Agency to require genetic testing before abacavir treatment. Abacavir binds to HLA-B*57:01 and alters the repertoire of presented peptides, resulting in the activation of autoimmunity. Previous studies employing computational approaches to investigate such DHRs have relied solely on a few crystallized tripartite structures, thus overlooking the full presented peptidome, leading to unsatisfactory predictive results. Here, we employed a state-of-the-art modeling approach to generate HLA structures complexed with over 13 000 presented peptides. We then established a novel computational modeling pipeline to simulate the binding of abacavir to these HLA-peptide complexes. Benchmarking against experimentally determined structures showed that this approach successfully recapitulated the crystalized tripartite structures with high accuracy (RMSD<2.2 Å). We then profiled alterations of the peptide repertoire at key positions in the presence of abacavir and proposed a method that accurately predicts compounds known to trigger T-cell activation. Overall, these results show that comprehensive modeling of the HLA-bound peptidome using advanced structural approaches can enhance the prediction and mechanistic understanding of immune-mediated DHRs.

## Graphical Abstract

**Graphical Abstract:**
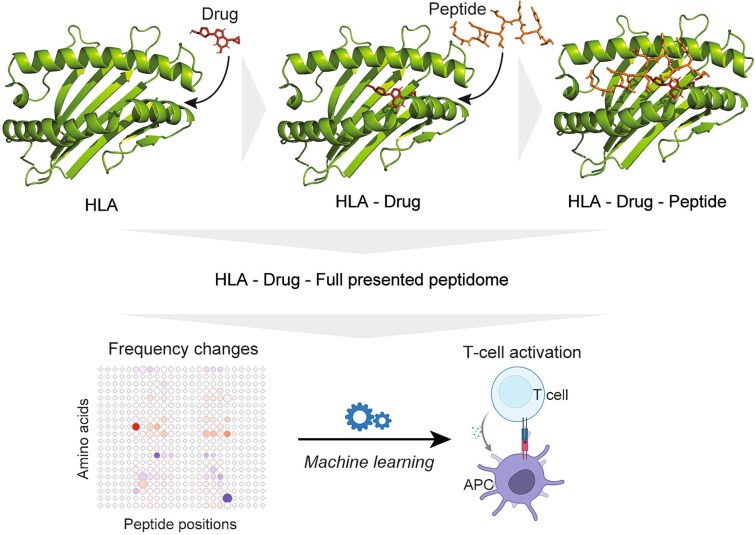

## Introduction

Drug hypersensitivity reactions (DHRs), defined as immune-mediated adverse drug reactions (ADRs) triggered by a wide range of medications, including antibiotics, antiepileptics, nonsteroidal anti-inflammatory drugs, have been identified as a key challenge in drug development and global health. Symptoms of DHRs can be systemic, commonly manifesting in the skin, but may also affect other organs such as the liver, lungs, kidneys, or heart [1]. Clinical presentations range from mild rashes to severe conditions such as Stevens–Johnson syndrome or toxic epidermal necrolysis, the latter carrying a mortality rate up to 50% [2]. Overall, DHRs account for one-third of all ADRs [3], which are the fourth to sixth leading cause of death and cost up to 136 billion USD annually in the US alone [4].

A significant proportion of DHRs are T cell-medicated (Type IV ADRs with delayed responses). In this type, human leukocyte antigens (HLAs) on antigen presenting cells present peptides to T cells, which can result in the activation of T cell-mediated immune responses. Importantly, *HLA* genes are highly polymorphic, resulting in differences in antigen presentation. Consequently, many *HLA* alleles are strongly associated with the risk of DHRs [1, 5]. The arguably most extensively studied example is the association between HLA-B*57:01 and abacavir hypersensitivity syndrome (ABC-HSS). Clinical evidence shows that around half of the HLA-B*57:01 carriers develop ABC-HSS following abacavir treatment [positive predictive value (PPV) = 47.9%], whereas non-carriers are fully protected [negative predictive value (NPV) = 100%] [6]. Considering this strong genetic predictive effect, both the US Food and Drug Administration (FDA) and European Medicines Agency (EMA) mandate HLA-B*57:01 screening prior to initiating or reinitiating abacavir treatment to prevent ABC-HSS. The mechanism underlying ABC-HSS has been elucidated by crystallography data, in which abacavir binds to the F pocket in HLA-B*57:01 and forms hydrogen bonds with Asp114 and Ser116, two amino acids that distinguish HLA-B*57:01 from other HLA-B variants, such as HLA-B*57:03 [7, 8]. The binding of abacavir alters the native peptide repertoire by ~20%–25%, disrupting self-recognition and leading to aberrant T-cell activation.

Using the resolved crystal structures of HLA-B*57:01 in complex with abacavir as templates, computational approaches such as molecular docking have been employed to elucidate HLA–drug interactions and predict HLA-B*57:01-associated DHRs [9]. However, the docking strategies used in these studies generally lack predictive power [10–13], mainly due to the following reasons: firstly, the HLA-B*57:01 structures used for modeling were derived from the HLA-B*57:01-abacavir complexes, i.e. the side-chain conformations within the binding site are already biased towards the abacavir-bound state rather than the naïve, unbound state. Secondly, past analyses considered less than three peptides or no peptide binding at all. Given the critical role and diversity of the presented peptide repertoire in the immunogenic process, incorporating the entire peptide repertoire rather than a limited subset is essential. Thirdly, the prediction of DHR risk in these studies was based solely on drug binding, whereas alterations in the presented peptide repertoire—the key mediator of autoimmunity—was disregarded.

To address these drawbacks, we here constructed 3D structures of the entire presented peptide repertoire of HLA-B*57:01 using an AlphaFold-derived modeling strategy at scale [14]. Starting from a naïve HLA structure, we established a distinct modeling pipeline that can accurately model all crystalized HLA-abacavir-peptide structure complexes, with root mean square deviations (RMSDs) below 2.2 Å compared to the experimentally determined tripartite structures. Based on this novel pipeline, we then profiled the alteration of the presented peptidome upon small-molecule ligand binding and quantified the number and frequency changes at each peptide position. Based on the changes of peptide repertoire, we developed a machine learning-based method that accurately predicted compounds triggering T-cell activation. These results show that HLA-drug-peptide structures can be reliably modeled at scale across the peptidome and thus represent, to date, the first computational analysis of HLA–drug interactions that considers the entire presented peptidome. Such comprehensive modeling can facilitate more reliable prediction of HLA-associated drug hypersensitivity and opens new avenues for studying the molecular basis underlying drug-induced autoimmunity.

## Materials and methods

### Molecular docking and structural modeling tools

For modeling, we employed different computational approaches for different purposes (Supplementary Table S1). The details regarding the use of the two primary docking tools are presented below:

Autodock Vina (Version 1.2.6) [15, 16] was employed to generate docking poses and affinities of abacavir binding to the HLA-B*57:01-peptide complexes. We used Scrubber (Version 0.1.1, https://github.com/forlilab/molscrub) to generate abacavir 3D structure from the SMILES (pH = 7.4) and used AutoDockTools (provided as part of MGLTools 1.5.7) [17] to further converted it to *.pdbqt* format using the python script *prepare_ligand4.py*. HLA-B*57:01-peptide complexes were prepared for docking by removing water and other cofactors, adding missing atoms and hydrogens, and assigning Gasteiger partial charges by ProDy (Version 2.4.0) [18]. The docking search box (grid) was centered on the middle point of the centroids of peptide and peptide-binding groove (the antigen-binding cleft formed by the α1 and α2 domains of the heavy chain), and its size was set as 30 × 20 × 20 Å to capture the entire HLA groove and peptide. The pose with the lowest binding energy of each HLA-B*57:01-peptide complex was selected for further analysis. In a later step, the binding energy reflecting the interaction between HLA-B*57:01 and each candidate compound from the modeled HLA-B*57:01-compound complex was quantified using the –score_only mode of AutoDock Vina.

AutoDock CrankPep (ADCP, version 1.1) [19] was used to dock peptides to the HLA-B*57:01-compound complexes. Peptides (ligands) were prepared by extracting their structures either from crystallized complexes or from TFold-predicted models. HLA-B*57:01-compound complexes (receptors) were obtained either from crystallized structures or from Chai-predicted models. Preprocessed receptors were then used to generate target files using AutoGridFR (AGFR, included in the AutoDockFR software suite, version 1.0) [20], and the parameter of -asv was set at 1.1 for the use of AutoSite (version 1.1) binding-site detection [21]. ADCP was run to predict binding poses and corresponding affinities by setting parameters of -N (runs of independent searches) at 20, -n (number of steps) at 1 000 000, and -rmsd (root-mean-square deviation cutoff) at 2. Poses with maximum cluster sizes in top 10 clusters of each peptide were saved together with their affinity for further analysis.

Other modeling tools were run according to the information (input preparation and running platform) provided in Supplementary Table S1. An overview of how these tools were used in this study was summarized in Supplementary Fig. S1.

### Evaluation metrics for benchmarking structural modeling performances

The root mean square deviations (RMSDs) between predicted structures and ground truth (crystallized structures) were used to assess the generalizability of TFold [14] and further evaluate the performance of five peptide docking tools (ADCP [19], HADDOCK [22], HPEPDOCK [23], CABS-dock [24], and GalaxyPepDock [25]). The centroid distances between abacavir on predicted structures and corresponding crystallized structures were calculated to evaluate the suitability of four computational tools for modeling the interaction between abacavir and HLA-B*57:01, including Chai [26], Boltz-2 [27], Autodock Vina, and diffDock [28]. Evaluation metrics of RMSD and centroid distance were calculated in PyMOL (version 3.1 Schrödinger, LLC.), and details of the benchmarking processes were provided in Supplementary Method.

### Assessing peptide binding based on ADCP simulation

Based on the binding energies of all predicted poses for each peptide by ADCP, we established the following method to determine whether a peptide is likely to bind:

For each peptide *p*, we denoted the set of ADCP binding energies of its docking poses by ${\left\{{E}_{p,j}\right\}}_{j=1}^{M_p}$, where ${M}_p$ is the total number of poses for peptide *p*. We sorted these energies in ascending order (more favorable energies first), and define the per-peptide mean of the top *K* (=10) energies as follows:


(1) \begin{equation*} \overline{E_p}=\frac{1}{K_p}\sum_{k=1}^{K_p}{E}_p^{(k)} \end{equation*}

where ${K}_p=\min \left(K,{M}_p\right)$. (If fewer than *K* poses are available for peptide *p*, the mean is taken over the available *K_p_* poses.)

We partitioned the peptide set by peptide length *L* (here $L\in \left\{8,9,\mathrm{10,11,12,13,14}\right\}$) and defined ${\mathcal{P}}_L$ being the set of peptides with length *L*. The length-specific threshold *T_L_* was defined as the median of the per-peptide top-K means within that length class:


(2) \begin{equation*} {T}_L= median\left(\left\{\overline{E_p}:p\in{\mathcal{P}}_L\right\}\right) \end{equation*}

Peptide was classified as putative binder for length *L* if the binding energy of its best pose (pose with maximum cluster size in top 10 clusters of each peptide) satisfied $\overline{E_p}\le{T}_L$.

This method is established based on the following rationale: (i) averaging over the top K poses for each peptide of the same length reduces noise arising from individual docking poses and ensures that the classification reflects representative low-energy binding configurations of each peptide, rather than a single pose that may be unstable or spurious; (ii) the median of the length-specific, per-peptide top-K mean energies provides a length-normalized and outlier-resistant threshold. For a given length *L*, peptides can exhibit substantial variation in absolute ADCP energies, and a small number of unusually strong or weak binders may strongly skew the distribution. By taking the median across all peptides of length *L*, the threshold is defined by the central tendency of that length class and is therefore less sensitive to extreme values than measures such as the mean or minimum. Overall, this procedure enables comparison of peptides of the same length on a common scale while avoiding *ad hoc*, peptide-specific cutoffs.

### Machine learning method

We used a support vector machine (SVM) model trained on binding energy of selected 366 peptides to predict T-cell activation of a candidate compound. The kernel of the SVM model was set to a radial basis function. The hyperparameters, including the cost parameter (C) and the kernel width parameter (σ), were optimized using the caret package in R (version 4.5.1).

## Results

### Modeling the presented peptidome of HLA-B*57:01

**Figure 1:**
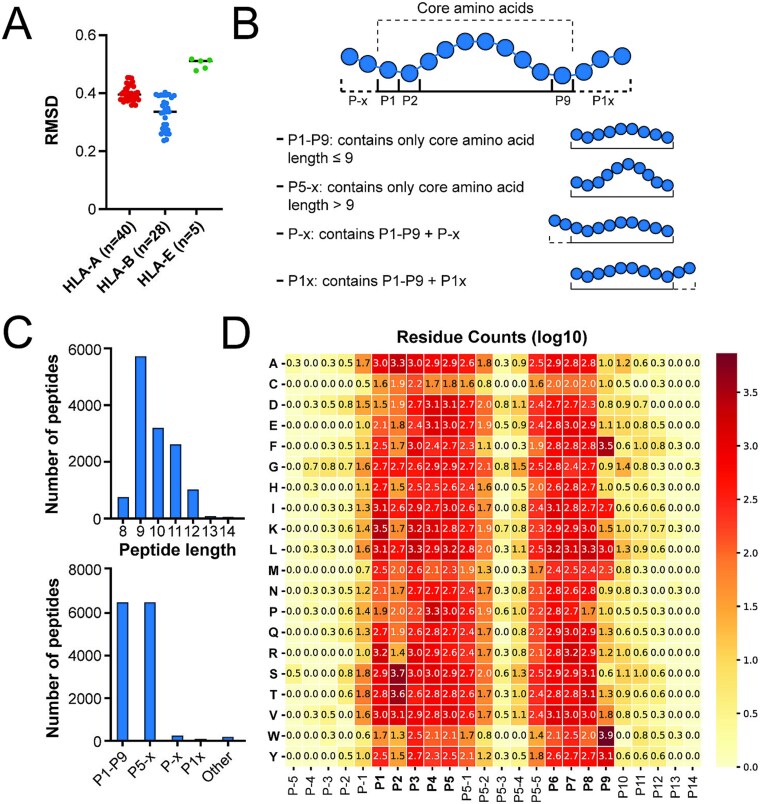
Panoramic view of the modeled HLA-B*57:01 presented peptidome. (A) Evaluation of modeling quality using 73 crystallized HLA-peptide complexes that were previously unseen by TFold. (B) Illustration of the alignment and categorization of predicted HLA-B*57:01-presented peptides based on the two anchor points, position 2 (P2) and position 9 (P9). (C) Distribution of a comprehensive repertoire of presented peptides (*n* = 13 706) across different lengths and categories. (D) Heatmap showing residue counts at each peptide position. Darker red shades represent more frequent occurrences of amino acids at the corresponding positions.

To understand immune ADRs, it is critical to understand the impact that drug binding has on the presented peptidome. Nevertheless, previous studies did not systematically consider peptides primarily because only a limited number of tripartite 3D structures were available. To bridge this gap, we first focused on HLA-B*57:01, the most extensively analyzed HLA allotype, to comprehensively model structures with the human peptidome based on the AlphaFold-derived modeling pipeline TFold, which was trained on nearly 1000 unique class I and class II HLA-peptide complex structures from the Protein Data Bank (PDB) [29]. To independently validate model accuracy, we collected all HLA-peptide crystal structures published after the release of TFold (Supplementary Table S2), none of which were seen during its training. We found that all newly identified complexes structures (*n* = 73) were accurately modeled by TFold (RMSD<2 Å), with most accurate predictions obtained for HLA-B-peptide complexes (Fig. 1A).

Encouraged by this result, we subsequently modeled structures of HLA-B*57:01 with a comprehensive repertoire of presented peptides (*n* = 13 706) obtained from published peptidomics data of naturally presented major histocompatibility complex (MHC) ligands [30]. Modeling of these complexes identified amino acids at each peptide position, including the two primary anchor points, position 2 (P2) and position 9 (P9), which allowed the classification of all peptides into four main categories: (i) short and flat peptides with a length ≤9 amino acids (P1-P9) and extended peptides with a length >9 amino acids that can be further subdivided into (ii) centrally extended peptides resulting in bulged binding (P5-x), (iii) N-terminally extended peptides (P-x), and (iv) C-terminally extended peptides (P1x; Fig. 1B). Across the HLA-bound peptidome, 9-mers are most prevalent, followed by 10-mer, 11-mer, 12-mer, and 8-mer peptides. Among peptide types, P1-P9 and P5-x are the most abundant peptide types (*n* = 6545, 47.8%; 6542, 47.7%, respectively), whereas all other types are rare (Fig. 1C). This corroborates the notion that the class I HLA peptide binding cleft is closed at both ends, thereby largely restricting bound peptides to either being short and flat, or to a center-bulged shape. We then mapped the residue signatures at each peptide position by profiling amino acid abundance (Fig. 1D). We identified strong enrichment of tryptophan and phenylalanine at P9, and serine and tryptophan at P2, which closely aligns with the *in vitro* data [7], confirming the robustness of this analytical approach.

### Assessing the interaction between abacavir and HLA-B*57:01-peptide complexes using molecular docking

**Figure 2:**
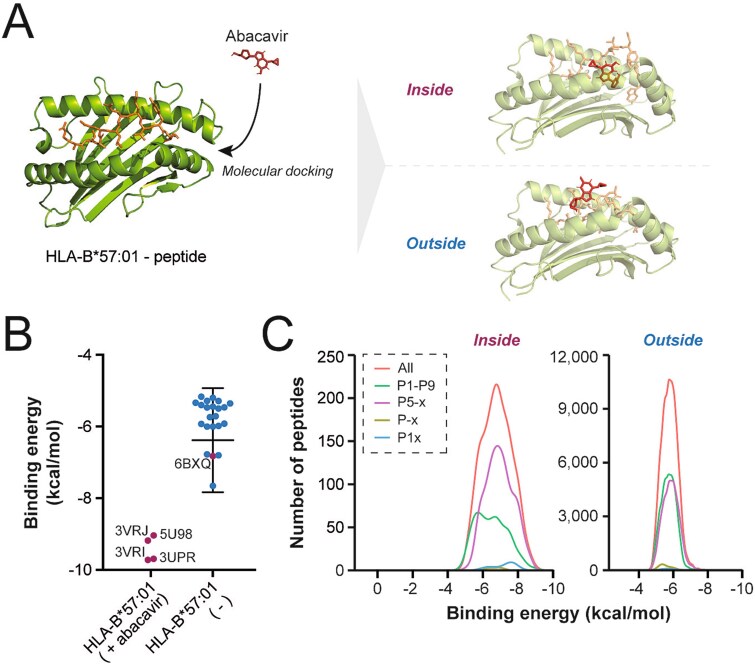
Assessment of the interaction between abacavir and HLA-B*57:01-peptide complexes using AutoDock Vina. (A) Schematic representation of the molecular docking configuration, with abacavir as the ligand and the HLA-B*57:01-peptide complexes as receptors. The resulted two types of binding positions were illustrated on the right side. (B) Distribution of binding energies obtained from docking abacavir to crystallized HLA-B*57:01-peptide complexes (“+ abacavir”: abacavir present in the original structure; “-”: no small-molecule ligand in the original structure). Abacavir molecules bound within the native binding pocket were indicated by PDB IDs. (C) Distribution of binding energies generated by docking abacavir to TFold-modeled HLA-B*57:01-peptide complexes across different peptide categories, divided according to the predicted abacavir binding positions.

Molecular docking has been widely employed to investigate HLA–drug interactions [9]. A commonly used strategy in previous studies was to dock drugs directly to either the unbound HLA structure or to HLA-peptide complexes, using the available crystal structures [12, 13]. Typically, crystal structures of HLA-B*57:01 complexed with abacavir were used to benchmark the docking protocol by removing abacavir and redocking it into the corresponding binding site. Following the same strategy, we first docked abacavir to all crystalized HLA-B*57:01-peptide structures available in the PDB, including the four abacavir-bound structures (3VRI, 3VRJ, 3UPR, and 5U98), from which abacavir was removed before redocking (Fig. 2A and B). We found that binding energies for abacavir-bound structures were significantly lower than in those that did not originally contain abacavir (Fig. 2B). These results suggest that redocking is biased by abacavir-favored binding site conformations and should be interpreted cautiously when used for benchmarking of docking protocols. Furthermore, upon inspecting the docking poses of abacavir, we observed that in structures that were crystallized without abacavir, the drug molecule was almost exclusively positioned on the protein surface rather than within the native binding pocket identified in the crystallized complexes. To investigate whether this phenomenon resulted from the limited number of bound peptides tested, we further docked abacavir to all TFold-generated HLA-B*57:01-peptide complex structures encompassing the entire peptide repertoire (Supplementary Fig. S1A). Similarly, only a very small fraction (<3%) of these complexes accommodated abacavir within the binding cleft (Fig. 2C). This finding contrasts with experimental evidence showing that 75%–80% of peptides are still presented in the presence of abacavir [7]. Combined, these results indicate that this commonly used docking strategy has limited capability to identify compounds exhibiting abacavir-like binding profiles.

### Establishment of a novel pipeline to model tripartite HLA complexes

**Figure 3:**
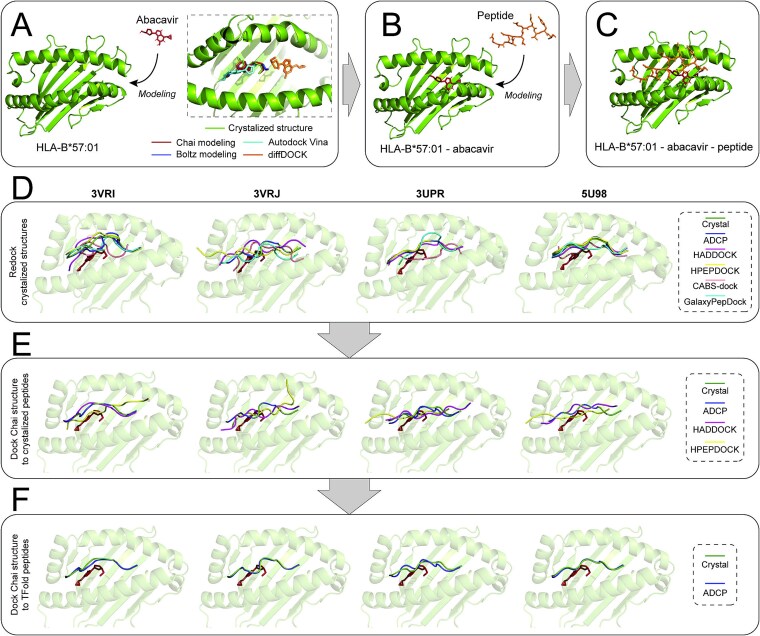
Establishment of a novel pipeline for modeling HLA-B*57:01-abacavir-peptide complexes. (A) Comparison of HLA-B*57:01-abacavir complexes generated using Chai, Boltz-2, AutoDock Vina, and diffDock. (B, C) Schematic representation of the docking procedure for placing peptides onto HLA-B*57:01-abacavir complexes. (D) Benchmarking of protein-peptide docking algorithms (AutoDock CrankPep, HADDOCK, HPEPDOCK, CABS-dock, and GalaxyPepDock) by redocking crystallized peptides to crystallized HLA-B57:01-abacavir complexes. (E) Benchmarking of top-performed protein-peptide docking algorithms (AutoDock CrankPep, HADDOCK, and HPEPDOCK) by docking crystallized peptide structures onto the HLA-B*57:01-abacavir complex generated by Chai. (F) Benchmarking of AutoDock CrankPep by docking TFold-generated peptide structures onto the Chai-generated HLA-B*57:01-abacavir complex.

The failure to dock abacavir to its native site is likely caused by peptides that obstruct the access pathway to the interior of the binding cleft. Therefore, we adopted an alternative strategy in which abacavir was first modeled to interact directly with the unbound HLA-B*57:01 structure, followed by peptide docking to the resulting HLA-B*57:01-abacavir complex (Fig. 3A–C; Supplementary Fig. S1B). In the first step, we compared the performance of three state-of-the-art biomolecular interaction predictors Chai [26], Boltz-2 [27], and diffDock [28], as well as arguably the most commonly used docking tool Autodock Vina [15, 16], to model the interaction between abacavir and HLA-B*57:01. While diffDock placed abacavir between the B and D pockets, the other three modeling approaches (Chai, Boltz-2, and AutoDock Vina) correctly predicted abacavir binding in the F pocket (Fig. 3A). The optimal docking pose generated by AutoDock Vina positioned abacavir vertically within the F pocket, in contrast to the nearly horizontal orientation observed in the native structure. As a result, the purine ring of abacavir was displaced away from the “floor” of the binding groove and was unable to form hydrogen bonds with Asp114 and Ser116. In comparison, the abacavir poses predicted by Chai and Boltz-2 closely resembled the native pose, with centroid distance below 1 Å (Supplementary Table S3). However, Boltz-2 modeled the hydroxyl group at the 1-position pointing in a direction orthogonal to the positioning in the crystal structure. We therefore selected Chai as the most accurate tool for modeling the interaction between abacavir and HLA-B*57:01. Notably, modeling of the abacavir-HLA-B*57:03 complex revealed an unfavorable binding energy of −3.49 kcal·mol^−1^, strongly suggesting that abacavir cannot stably bind to HLA-B*57:03 in the absence of key mutations at positions 114 and 116, in agreement with experimental findings [7].

In the next step, we performed systematic docking experiments to benchmark the suitability of five protein-peptide docking algorithms, namely AutoDock CrankPep (ADCP) [19], HADDOCK [22], HPEPDOCK [23], CABS-dock [24], and GalaxyPepDock [25], to predict the binding of presented peptides to HLA-B*57:01-abacavir complexes, using the four available high-resolution crystalized structures (3VRI, 3VRJ, 3UPR, and 5U98; Table 1). First, we removed the binding peptides from the four structures and then redocked them. Both CABS-dock and GalaxyPepDock placed the peptides in horizontally rotated orientations that deviated from the native binding direction. HADDOCK and HPEPDOCK produced accurate poses for 3UPR and 5U98, but not for 3VRI and 3VRJ. In contrast, ADCP consistently predicted peptide poses that closely aligned with the native peptides across all four structures (RMSD≤2.1 Å; Fig. 3D). We then evaluated the higher-performing docking methods (ADCP, HADDOCK, and HPEPDOCK) by conducting docking using the Chai-generated HLA-B*57:01-abacavir structure rather than the experimentally determined ones. The results showed that only ADCP could accurately predict peptide positions across all structures (RMSD≤2.2 Å; Fig. 3E). To test whether ADCP also handle peptide structures generated by TFold, we further performed docking using the Chai-generated HLA-B*57:01-abacavir structure and the four peptides derived from TFold-generated models. The results showed that ADCP successfully predicted the binding poses of all TFold-generated peptides (Fig. 3F). We thus concluded that Chai-modeled small molecules binding followed by ADCP modeling of peptide binding constitutes a reliable and scalable workflow to construct tripartite HLA-B*57:01-abacavir-peptide complexes.

**Table 1:** Benchmarking five protein-peptide docking algorithms.

**Docking mode**	**Structures (PDB ID)**	**Peptide poses from different docking tools compared with crystalized structures (in Å)**				
		***ADCP***	***HADDOCK***	***HPEPDOCK***	***CABS-dock***	***GalaxyPepDock***
Redocking	3VRI	2.1	3.6	1.0	6.5	3.6
	3VRJ	1.2	6.5	6.4	15.4	3.0
	3UPR	1.0	1.3	0.5	15.4	2.8
	5U98	1.5	1.3	0.7	14.8	2.8
Docking to the Chai structure	3VRI	1.4	1.3	18.9	/	
	3VRJ	1.7	15.8	15.5	/	
	3UPR	2.2	15.7	5.1	/	
	5U98	1.5	15.7	6.3	/	
Docking to the Chai structure using peptides from the TFold	3VRI	2.1	/			
	3VRJ	1.8	/			
	3UPR	2.6	/			
	5U98	1.6	/			

### Impact of abacavir binding on the presented peptidome

This newly established pipeline enabled the systematic modeling of HLA-B*57:01-abacavir complex interactions with the entire human peptide repertoire. Peptides were grouped by length, and within each group, their binding was determined based on the spectrum of binding energies across all generated docking poses for each peptide (see Methods). Overall, 63.9% (*n* = 8753) of all peptides were predicted to still bind HLA-B*57:01 in the presence of abacavir, in agreement with the previously reported findings [7]. Binding energy exhibited a positive correlation with peptide length (Supplementary Fig. S2).

**Figure 4:**
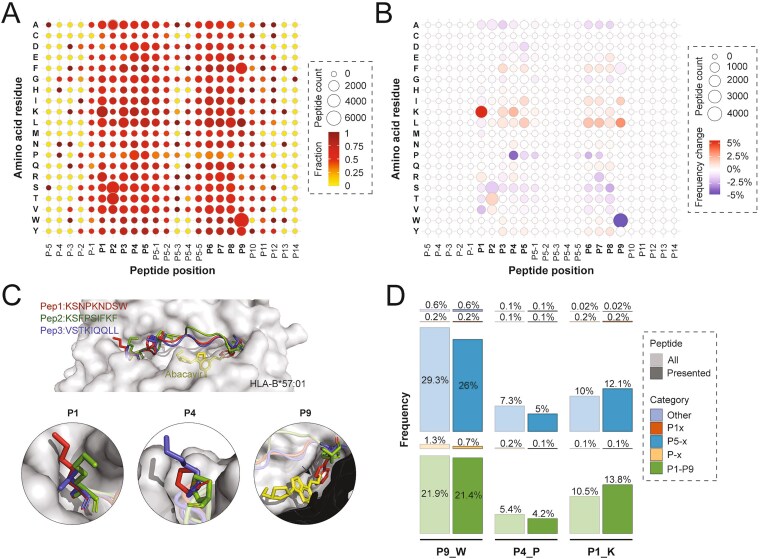
Alteration of the peptide repertoire induced by abacavir binding to HLA-B*57:01. (A) Bubble heatmap showing the proportion of bound amino acid residues at each peptide position. Increased intensity indicates a higher ratio of peptides containing the corresponding residue that can bind to the HLA-B*57:01-abacavir complex, while larger circles represent a greater number of peptides with that residue in the overall HLA-B*57:01-presented peptidome. (B) Bubble heatmap showing changes in residue frequencies at each peptide position compared with the original HLA-B*57:01-presented repertoire. Darker (redder or bluer) shades indicate larger differences in residue frequency between peptides bound in the presence or absence of abacavir, while larger circles indicate higher absolute bound peptide counts for that residue at the given position. (C) Structural illustration of three peptides (KSNPKNDSW, KSFPSIFKF, and VSTKIQQLL) interacting with the HLA-B*57:01-abacavir complex at positions P1, P4, and P9, showed in overall and zoomed-out view. The arrow at P9 (zoomed-out view) indicates the region where structural clashes may occur. (D) Frequency of tryptophan at P9, proline at P4, and lysine at P1 in HLA-B*57:01-abacavir-bound peptides compared with the original HLA-B*57:01-presented peptides, with colors indicating different peptide categories.

Comparing the change in peptide repertoire upon abacavir binding, we found that lysine (K) at P1 was increasingly enriched in abacavir bound structures, while isoleucine (I) and leucine (L) were overrepresented across peptide positions (Fig. 4A and B). In contrast, tryptophan (W) and proline (P) were significantly decreased at P9 and P4, respectively. To obtain structural insights into these observed signatures, we analyzed representative TFold-generated structures containing peptides that either do not bind (pep1, KSNPKNDSW) or bind (pep2, KSFPSIFKF and pep3, VSTKIQQLL) HLA-B*57:01 in the presence of abacavir (Fig. 4C). At the C-terminal P9, the indole ring of tryptophan in pep1 formed pronounced steric clashes with the cyclopropyl ring of abacavir, thereby preventing stable accommodation of tryptophan and explaining why this amino acid is not favored at P9 in the presence of abacavir. At the N-terminal P1, lysine was more favored likely due to its flexible four-methylene-group side chain that can fits well against the “wall” of the binding groove formed by the A pocket, particularly in the presence of abacavir, which narrows the peptide-binding site. At P4, while the binding of abacavir only marginally shifted the position of the pyrrolidine ring in proline, as indicated by the differences between pep1 and pep2, the flexible lysine was slightly more preferred. Indeed, we found that among peptides containing lysine at P1 but not P4 (*n* = 2662), at P4 but not P1 (*n* = 1041), or at both P1 and P4 (*n* = 183), the majority (79.4%, 76.4%, and 96.2%, respectively) can bind to the HLA-B*57:01-abacavir complex, indicating that the presence of lysine at P1 and/or P4 favors peptide binding in the presence of abacavir. Furthermore, within the binding peptides, lysine is more frequently present at either P1 or P4 alone, suggesting that when abacavir binds, lysine at one site may confer the conformational flexibility necessary to tolerate variation at the other. These mechanistic interpretations were further supported by a broader analysis of contact numbers and ADCP affinities for key residues at P1, P4, and P9 across all peptides (Supplementary Table S4).

We then examined the peptide sources of the frequency changes of tryptophan at P9, proline at P4, and lysine at P1 (Fig. 4D). We found that the decreased frequency of tryptophan at P9 was predominantly driven by the center-bulged peptides (P5-x), suggesting that longer peptides with a bulged center are more likely to have the C-terminal residues inserted deeply into the F pocket, thereby colliding with abacavir when tryptophan occupies the P9 position. In contrast, the increased frequency of lysine at P1 was mainly due to the short and flat peptides (P1-P9), indicating that when the peptides fit more closely to the HLA molecule (in case of P1-P9 peptides), adjustments of the N-terminal residues become more necessary in the presence of abacavir within the binding cleft.

### Peptidome-based prediction of drug-induced hypersensitivity risk

**Figure 5:**
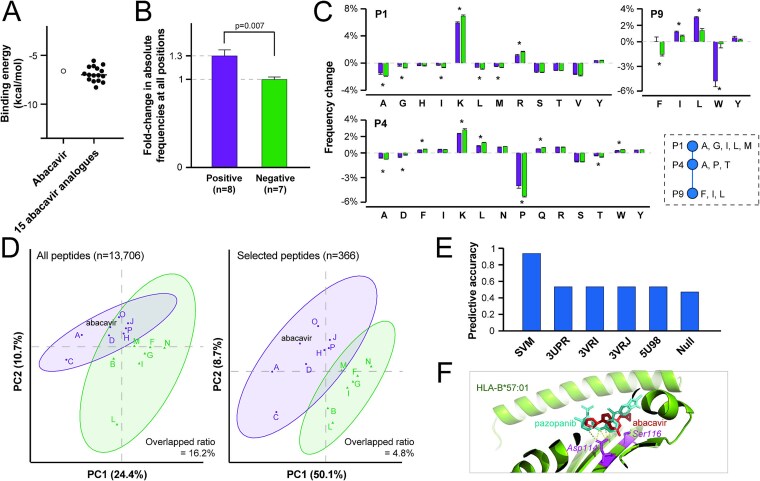
Alterations of the peptide repertoire can predict T-cell activation. (A) Distribution of calculated binding energies for HLA-B*57:01-abacavir analogue complexes modeled by Chai. (B) Comparison of peptide count-weighted frequency fold-change between positive and negative abacavir analogues. (C) Amino acid residues showing significant frequency changes at positions P1, P4, and P9, resulting in positive-favored motifs at each position (i.e. alanine, glycine, isoleucine, leucine, and methionine at P1; alanine, proline, and threonine at P4; and phenylalanine, isoleucine, and leucine at P9). (D) Principal component analysis (PCA) plots with 95% confidence ellipses based on binding energies of all peptides (*n* = 13 706) in the HLA-B*57:01-presented repertoire and of peptides (*n* = 366) selected based on positive-favored patterns. Abacavir analogues were annotated based on labels from the previous publication [31]. (E) Comparison of predictive accuracy between the established SVM-based model and conventional methods using single abacavir-bound structures. (F) Chai-modeled pazopanib binding pose in comparison with abacavir within the HLA-B*57:01 binding cleft. Predicted hydrogen bonds were indicated in dash lines.

We next investigated whether drug-induced changes in the peptide repertoire could predict compounds that induced immune activation. To this end, we focused on 15 compounds with well-characterized HLA-based *in vitro* T-cell activation profiles [31]. These compounds were generated by modifying the cyclopropyl moiety of abacavir, a chemical group previously shown to be critical for T-cell activation [32, 33]. As a first step, we modeled the interaction of HLA-B*57:01 with these compounds using Chai. All 15 compounds adopted binding poses closely aligned with the native abacavir pose (RMSD<2 Å) and showed binding energies similar to abacavir (Fig. 5A).

Next, we analyzed the frequency changes of amino acids at each peptide positions induced by the tested abacavir analogues. After weighting by peptide counts, we found that the overall peptide frequencies were significantly changed (1.3-fold increase) for positive analogues compared to negative ones (Fig. 5B). Examination of the amino acid frequency changes at P1, P4, and P9, positions identified as critical for peptide-HLA binding, revealed significant differences between the binding patterns of immunogenic and non-immunogenic compounds (Fig. 5C). We then evaluated peptide binding signatures systematically and found that positive and negative abacavir analogues could be distinguished based on their binding energies across the entire repertoire of 13 706 peptides (Fig. 5D, left panel). To enhance classification performance and reduce the number of peptides for more efficient large-scale screening, we focused on peptides that were enriched in immunogenic compounds (i.e. peptides containing amino acids with significantly positive frequency changes at P1, P4, and P9, shown in Fig. 5C), resulting in a refined subset of 366 peptides (Supplementary Table S5). Using this peptide subset, positive and negative abacavir analogues could be clearly distinguished (Fig. 5D, right panel).

To generate binary predictions, we then used the binding energies of these 366 peptides (prior knowledge derived from the statistical results in Fig. 5C) to train an SVM model and performed leave-one-out cross-validation on the 15 abacavir analogues. The resulted model achieved 88% sensitivity and 100% specificity in distinguishing positive from negative analogues. In contrast, previous approaches that evaluated compound docking to four crystallized structures containing only a single peptide (3VRI, 3VRJ, 3UPR, and 5U98) or that ignored peptide binding (null), and relied on empirically defined binding energy thresholds (−6.5 kcal·mol^−1^), exhibited substantially lower accuracy (Fig. 5E). To circumvent the arbitrariness arising from the use of empirically defined binding energy thresholds, we trained and cross-validated the same model using energy data from the binding of a single peptide, no peptide and a combined dataset (4 + null). The resulted algorithms preformed the best when trained on all 366 peptides, followed by the one trained on the combined dataset (Supplementary Table S6).

So far, HLA-B*57:01 has been associated with adverse reactions to only three drugs: abacavir [6], flucloxacillin [34, 35], and pazopanib [36]. While the mechanisms underlying abacavir ADRs have been evaluated in detail, the molecular bases for flucloxacillin- and pazopanib-induced reactions remain unclear. Flucloxacillin, a β-lactam antibiotic and a well-known cause of drug-induced liver injury (DILI), has been associated in genome-wide association studies with the HLA-B*57:01 allele, with reported odds ratios of up to 80 [34, 35]. Despite the absence of experimentally derived crystal structures, *in vitro* evidence showed that flucloxacillin can covalently bind to proteins within immune cells and form flucloxacillin-modified peptides [37]. Furthermore, the extent of this binding correlated with T-cell activation, strongly suggesting that flucloxacillin induces DILI via a hapten-mediated mechanism [38]. When modeled with HLA-B*57:01 using Chai, flucloxacillin showed relatively weak binding (binding energy = −5.9 kcal·mol^−1^). Furthermore, the predicted binding pose was evidently distinct from that of abacavir (centroid distance = 6.3 Å), indicating that flucloxacillin likely activates T cells via a mechanism different from abacavir in the altered peptide repertoire model (Supplementary Fig. S3).

Similarly to flucloxacillin, pazopanib, an effective treatment for advanced renal cell carcinoma, has been reported to confer a higher risk of liver injury in patients carrying the HLA-B*57:01 allele [36]. Previous docking analyses predicted that pazopanib occupies a binding pocket similar to that of abacavir and forms a hydrogen bond with Asp114 of HLA-B*57:01, one of the two key residues (Asp114 and Ser116) mediating abacavir binding [36]. In our analysis, pazopanib exhibited a binding energy (−8.07 kcal·mol^−1^) and binding pose (centroid distance = 1.4 Å) comparable to those of abacavir. Consistent with the previous study, our modeling showed that pazopanib forms hydrogen bonds with Asp114, but not with Ser116 (Fig. 5F). When modeled with HLA-B*57:03, the binding pose shifted significantly (centroid distance = 5.90 Å), confirming the importance of Asp114 as one of the key amino acid differences between HLA-B*57:03 and HLA-B*57:01. Next, we docked the subset containing 366 peptides to the HLA-B*57:01-pazopanib structure. Our SVM-based algorithm correctly predicted pazopanib as a drug with DHR risk. Mechanistically, pazopanib is a larger molecule than abacavir and occupies a significant space towards the F-pocket of HLA-B*57:01. This binding mode is expected to have a strong impact on peptide representation. Our results provide proof-of-concept that the strategy developed here can predict HLA-associated hypersensitivity risk not only for abacavir analogues but also for structurally distinct compounds.

## Discussion

HLA-mediated DHRs are among the most clinically significant ADRs due to their association with a wide range of medications and their potentially fatal outcomes [39]. While they constitute a major focus in pharmacogenomics, the complexity of their mechanisms and the influence of multiple confounding factors hinder the clinical translation of genetic HLA biomarkers [40]. A key hurdle for successful modeling and prediction of DHRs lies in the fact that the peptide repertoire, the critical component driving T-cell activation, could not be accurately captured in previous models [10–13, 31]. In this context, the standard approach is to dock compounds into the available crystallized HLA-B*57:01-abacavir-peptide structures, either with abacavir removed or with both abacavir and the peptide removed. The risk of DHRs is then assessed based on the predicted binding confidence of the compounds, under the hypothesis that strong binding could reshape the binding groove and thereby alter the peptide repertoire [10–13, 31]. In recent years, the structural modeling of proteins and their interactions with diverse ligands has greatly advanced by state-of-the-art machine learning models, with the success of AlphaFold serving as a prime example [41, 42]. The emergence of AlphaFold further facilitated the development of TFold, an AlphaFold-based pipeline for modeling structures of HLA-peptide complexes using 928 unique class I and class II complexes from the PDB [14]. These recent advances in structural biology thus provide a timely opportunity to comprehensively characterize the drug–HLA interaction in the presence of the complete presented peptide repertoire.

In light of this, we generated structural models of HLA-B*57:01 in complex with more than 13 000 binding peptides using TFold. By applying the same docking approach as previous studies, but instead using a naïve HLA structure binding with entire peptide repertoire, we demonstrated the importance of considering the full peptidome in HLA–drug interaction analyses, which may explain the limited predictive performance of earlier studies [9]. Subsequently, we established a modeling pipeline that, for the first time, enables systematic investigation of how drug binding alters the peptide repertoire. In this pipeline, three AI-based generative models (Chai, Boltz-2, and diffDock) and one docking program (Autodock Vina) were used to simulate HLA-B*57:01-abacavir interaction. Chai and Boltz-2 are general biomolecular interaction modeling tools that can predict the 3D structures of a much wider range of complexes, including protein–protein, protein–ligand, and protein–nucleic acid interactions [27]. In contrast, the prime task of diffDock is to solve the protein-small-molecule ligand docking problem [28]. Autodock Vina, on the other hand, is a traditional molecular docking algorithm that predict binding poses of small molecules and generate estimated binding energy scores using a physics-based searching and scoring function [15]. When predicting interactions between HLA-B*57:01 and abacavir, the advantage of using AI-based modeling is that predictions can leverage both the known structure of the binding cleft and the vast number of interaction patterns that have been learned from prior data. Furthermore, the side-chain conformations within the binding groove can be modeled more accurately using generative models [14]. In contrast, physics/chemistry-based docking methods conduct *de novo* evaluations of ligand binding poses and may exhibit improved performance for previously unseen chemotypes. However, these approaches generally assume a rigid or semi-flexible protein structure, limiting their ability to accurately capture side-chain conformational changes upon ligand binding [43]. Notably, most AI-based modeling methods cannot properly distinguish between chiral compounds, as enantiomers are often treated identically. In contrast, traditional physics-based docking methods can explicitly account for molecular chirality during binding pose evaluation.

To further evaluate peptides binding to the HLA-B*57:01-abacavir structure, we benchmarked five algorithms for modeling of protein–peptide interactions (ADCP, HADDOCK, HPEPDOCK, CABS-dock, and GalaxyPepDock) using the four PDB-derived structures (3VRI, 3VRJ, 3UPR, and 5U98). Among these programs, only GalaxyPepDock is template-based, relying on the structures of similar complexes for its predictions [25]. In contrast, template-free methods comprise CABS-dock, which performs global docking; ADCP and HADDOCK, which perform local docking; and HEPEDOCK, which is capable of both global and local docking. This explains that with a predefined HLA binding groove (see Methods), ADCP, HADDOCK, and HEPEDOCK performed significantly better than CABS-dock (Fig. 3D). The five docking programs have previously been evaluated using a universal benchmark set comprising 185 protein-peptide complexes with peptide length ranging from 5 to 20 residues [44]. With a predefined binding site, ADCP achieved the best prediction performance, in agreement with the results of our benchmark experiment.

Based on the established pipeline, we identified 8753 of 13 706 peptides that bound to HLA-B*57:01 in the presence of abacavir, thereby allowing us to capture alterations of the presented peptide repertoire. Notably, this analysis was based on the peptide repertoire presented by HLA-B*57:01 in the absence of abacavir. Therefore, novel peptides presented by HLA-B*57:01 in the presence of abacavir cannot be determined. However, even without considering *de novo* peptide binding, we found that amino acid frequency changes at different peptide positions mirrored *in vitro* results [7], suggesting that analysis based on the original peptide repertoire is sufficient to capture drug-induced peptide changes.

To explore whether the peptide repertoire alteration could be used to predict T-cell activation, we tested 15 abacavir analogues with well-characterized immunogenicity *in vitro*. We observed significantly greater changes in the bound peptidome for immunogenic analogues compared to negative compounds, indicating that alteration of the peptide repertoire can serve as a strong predictor of T-cell-activating potential. This finding opens new avenues for employing computational modeling approaches to predict HLA-mediated DHRs at scale, offering more accessible alternatives to conventional cost- and time-consuming lymphocyte transformation tests [45]. To increase computational efficiency, we generated a subset of 366 peptides based on altered amino acid signatures at key peptide positions. This panel reduced computational time and costs from ~7300 CPU core hours to about 200 core hours per candidate compound. Of note, this peptide panel can only be used for prediction of HLA-B*57:01-associated DHRs and peptide panels for other allotypes of interest need to be determined in the future. So far, only a few drugs have been linked to HLA-B*57:01-mediated hypersensitivity reactions. While we have demonstrated the predictive power of our modeling approach on these drugs, the same method could be applied in the future to HLA alleles with a larger number of clinically associated drugs, such as HLA-A*33:01 [46], to elucidate their potential mechanisms underlying DHRs.

While our data demonstrate that leveraging state-of-the-art computational methods can effectively model HLA-drug-peptide structures and enhance DHR prediction, this study has several limitations. First, as noted above, our analysis of peptide repertoire alteration was limited to peptides presented in the absence of abacavir and did not account for the *de novo* peptides presented upon drug binding (~20% of the repertoire in case of abacavir [7]). Although previous evidence suggests that these *de novo* peptides follow a broadly similar pattern at each peptide position [31], we cannot exclude the possibility that compound binding induces the presentation of peptides that substantially alter these patterns, potentially affecting the accuracy of the established prediction strategy. Second, the machine learning-based method for immune activation prediction was developed using a relatively small set of experimentally characterized compounds. More rigorous benchmarking and additional validation using independent datasets, particularly those comprising chemical structures largely distinct from abacavir, would improve the robustness of the current approach. Third, the HLA-drug-peptide modeling pipeline, as well as the subsequent T-cell activation prediction, were developed based on the altered peptide repertoire model, in which drugs directly bind to the HLA variant within the peptide-binding groove, with HLA-B*57:01 and abacavir serving as a well-established example. Therefore, this approach is suitable for investigating HLA variant-associated DHRs under this model, but is not applicable to DHRs triggered by other mechanisms, such as the hapten model, where drugs covalently bind to peptides, or the pharmacological interaction (p-i) model, in which drugs interact noncovalently with HLA-peptide complexes and/or T cell receptors [40]. While these mechanisms have been implicated in hypersensitivity reactions induced by a range of drugs, including amoxicillin, flucloxacillin, carbamazepine, and allopurinol [40], further computational modeling strategies are required to enhance prediction of immune-mediated DHRs arising from different mechanisms.

## Conclusion

In summary, this study provides a novel pipeline for the comprehensive modeling of drug-induced changes to the HLA-bound peptidome. The findings reveal how drug binding can alter the peptide presentation of antigen presenting cells and how such alteration can provide the molecular basis for drug-induced hypersensitivity. The established pipeline for modeling HLA-B*57:01-drug-peptide complexes, along with the 366 identified peptides, enables large-scale screening of immunogenic compounds and provides a proof-of-concept that the incorporation of presented peptidome through advanced structural modeling can refine the prediction of HLA-associated DHRs.

**Key Points**

- The full presented peptidome has a significant impact on HLA-mediated drug hypersensitivity reactions (DHRs), yet this factor has been largely overlooked in previous computational studies of DHRs.
- We established a novel computational modeling pipeline to generate HLA-drug-peptide tripartite structures.
- Modeling drug-induced alterations in the presented peptide repertoire enhances both the prediction and mechanistic understanding of immune-mediated DHRs.

## Supplementary Material

Supplementary_Figure_1_bbag350

Supplementary_Figure_2_bbag350

Supplementary_Figure_3_bbag350

Supplementary_Information_bbag350

Supplementary_Figure_legends_bbag350

## Data Availability

Relevant data supporting the key findings of this study are available within the article and the Supplementary Information files. All datasets, source codes, experimental results generated by the proposed method, and modeled structural complexes are available at https://github.com/Arvin-ZhongYi/HLA_BIB and https://doi.org/10.5281/zenodo.17456309.

## Notes

Biographical Note

Yitian Zhou is a researcher at Linköping University focusing on pharmacogenomics and computational modeling to study the impact of genetic variations on drug responses and diseases.
