# Supplementary figures and images for: Prediction of drug hypersensitivity by comprehensive modeling of HLA-peptidomes
Source: Brief Bioinform. 2026 Jul 3;27(4):bbag350. doi: 10.1093/bib/bbag350 (PMC13331351; doi:10.1093/bib/bbag350)

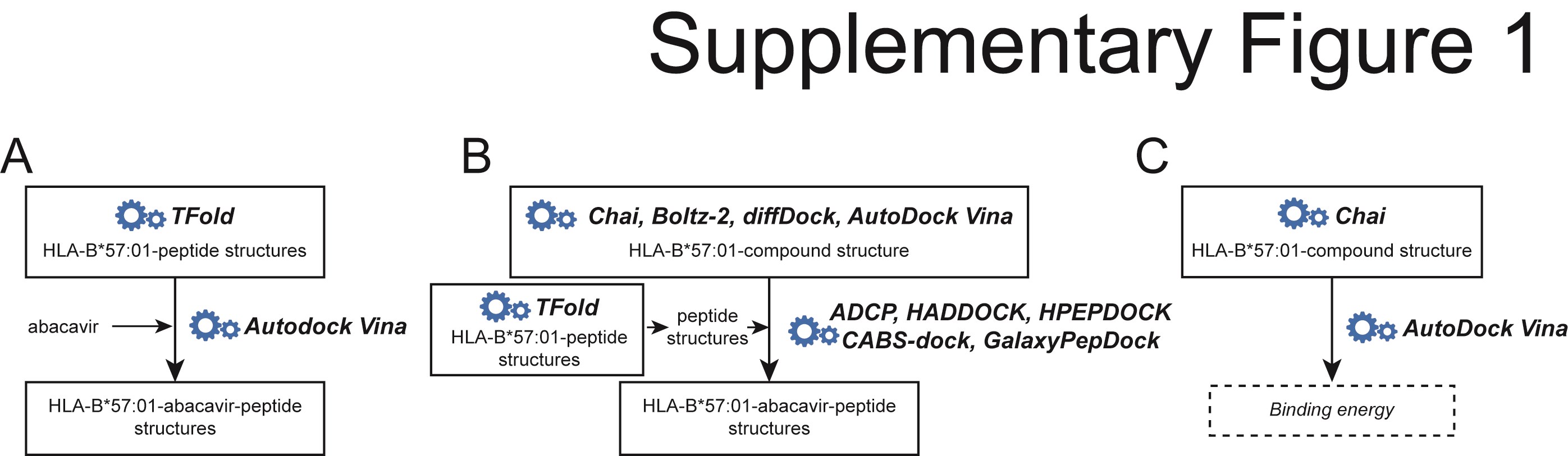

Supplement: Supplementary_Figure_1_bbag350 [file supplementary_figure_1_bbag350.jpeg]

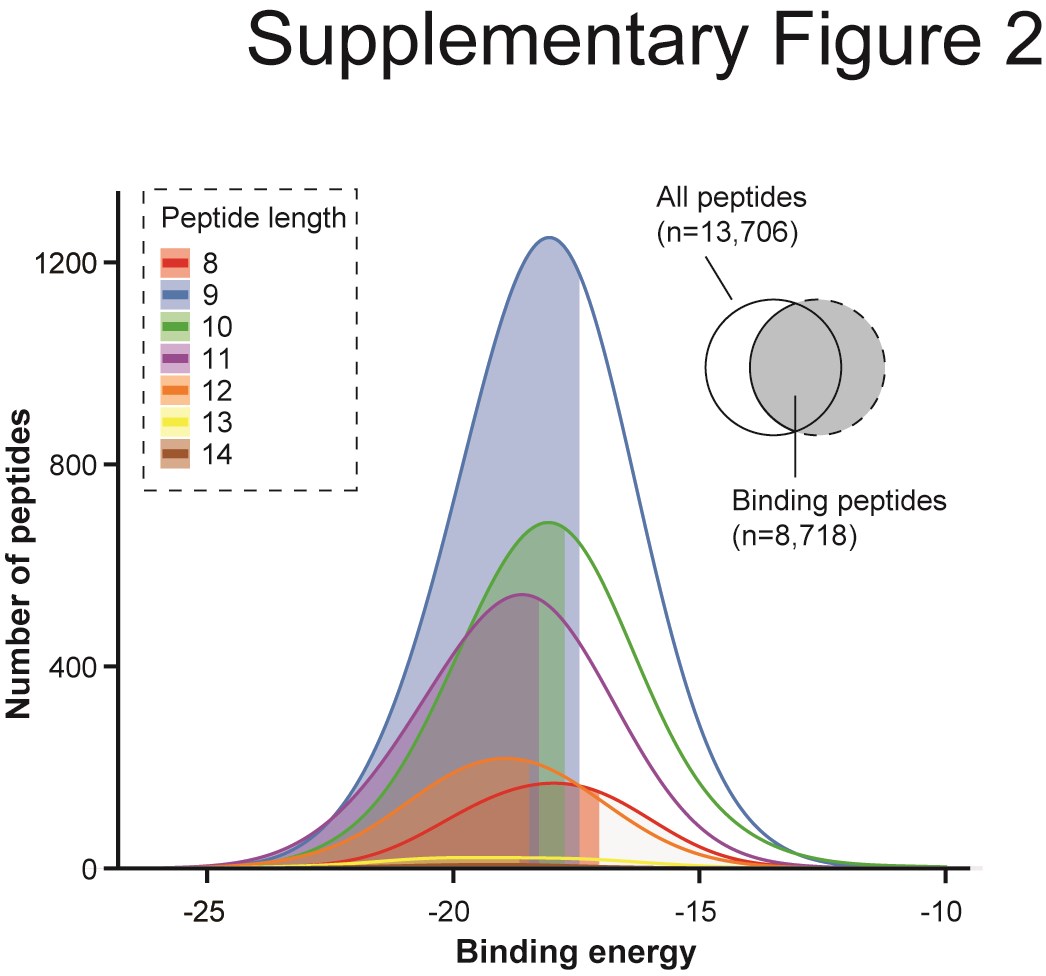

Supplement: Supplementary_Figure_2_bbag350 [file supplementary_figure_2_bbag350.jpeg]

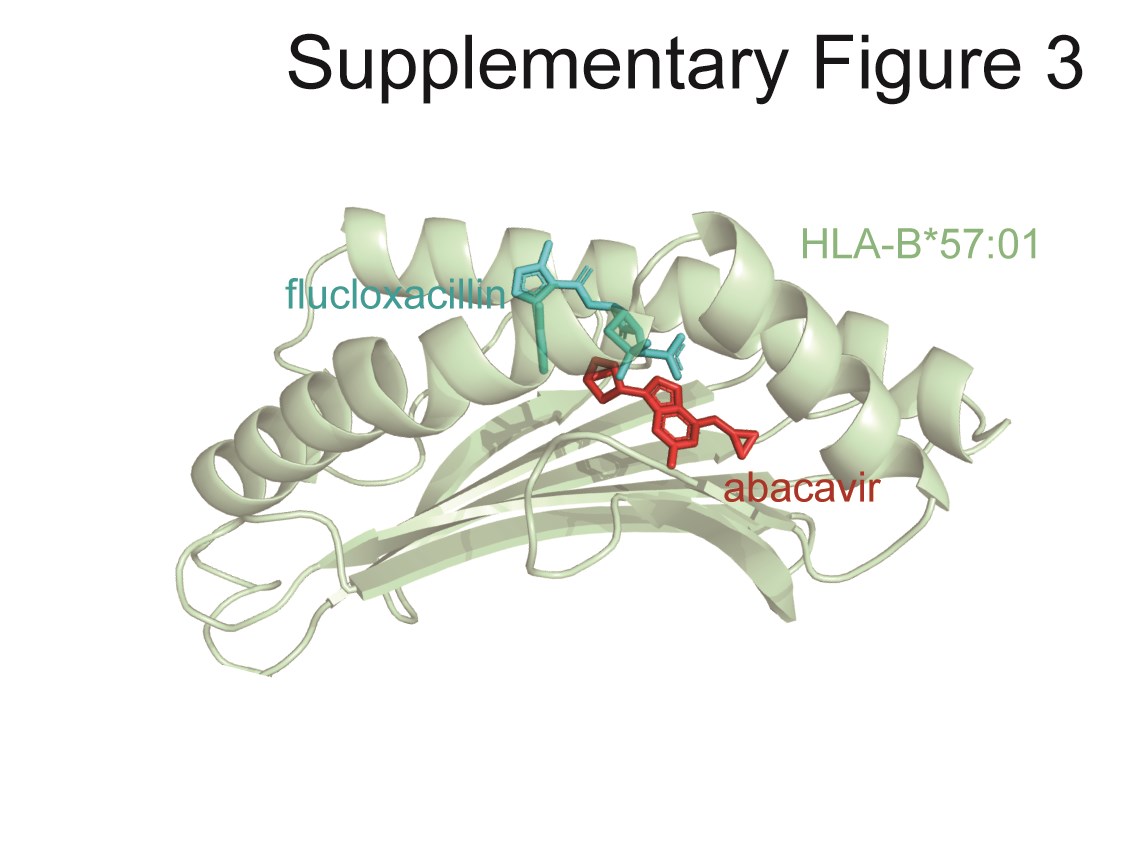

Supplement: Supplementary_Figure_3_bbag350 [file supplementary_figure_3_bbag350.jpeg]
