# Supplementary material for: Prediction of drug hypersensitivity by comprehensive modeling of HLA-peptidomes
Source: Brief Bioinform. 2026 Jul 3;27(4):bbag350. doi: 10.1093/bib/bbag350 (PMC13331351; doi:10.1093/bib/bbag350)
Supplement: Supplementary_Information_bbag350 [file supplementary_information_bbag350.pdf]

Methods

Evaluation metrics for benchmarking structural modeling performances

To assess the generalizability of TFold, we compared the predicted structures with the ground truth using RMSD (Equation 1), which was calculated by PyMOL (version 3.1 Schrödinger, LLC.).

$$RMSD = \sqrt{\frac{1}{N}\sum_{i=1}^{N}[(x_i - x'_i)^2 + (y_i - y'_i)^2 + (z_i - z'_i)^2]} \qquad \text{Equation 1}$$

where N is the number of equivalent atoms, and (x_i, y_i, z_i) and (x'_i, y'_i, z'_i) are the Cartesian coordinates of atom i in the reference and compared conformations, respectively. Prior to RMSD computation, the two structures were optimally superimposed by least-squares fitting to remove overall translational and rotational differences.

To evaluate the suitability of four computational tools for modelling the interaction between abacavir and HLA-B*57:01, including Chai [1], Boltz-2 [2], Autodock Vina and diffDock [3], we used four crystallized HLA-B*57:01-abacavir-peptide structures available in PDB (3VRI, 3VRJ, 3UPR and 5U98) as the ground truth structures. The HLA-B*57:01 binding groove from each structure was pre-processed as receptor after removing peptide and ligand from original PDB structure. Redocked HLA-B*57:01-abacavir poses from AutoDock Vina based on these four receptors, as well as modeled HLA-B*57:01-abacavir structures from Chai, Boltz-2 and DiffDock, were generated following the procedures described above. Centroid distances (Equation 2) between abacavir on predicted structures and corresponding crystallized structures was calculated in PyMOL (version 3.1 Schrödinger, LLC.) as the evaluation metric of suitability.

$$d = \|C_1 - C_2\| = \sqrt{(x_1 - x_2)^2 + (y_1 - y_2)^2 + (z_1 - z_2)^2} \qquad \text{Equation 2}$$

where $C_1 = (x_1, y_1, z_1)$ and $C_2 = (x_2, y_2, z_2)$ represent the Cartesian coordinates of the geometric centroids (Equation 3) of abacavir on predicted structure and crystalized structure respectively.

$$C = \frac{1}{N}\sum_{i=1}^{N} r_i \qquad \text{Equation 3}$$

where N is the number of heavy atoms in abacavir, and $r_i = (x_i, y_i, z_i)$ represents the Cartesian coordinates of atom i.

To further evaluate the performance of five docking tools (ADCP [4], HADDOCK [5], HPEPDOCK [6], CABS-dock [7] and GalaxyPepDock [8]) in predicting peptides interacting with the HLA-B*57:01-abacavir structure, we benchmarked them in three rounds using four

available crystal complexes (3VRI, 3VRJ, 3UPR, and 5U98) as ground truth and the RMSD (Equation 1) of peptide atoms as the evaluation metric. All docking tasks were executed following corresponding procedures described previously, and RMSDs were calculated in PyMOL (version 3.1 Schrödinger, LLC.).

Supplementary Tables

Table S1. Computational tools for Molecular docking and structural modeling

Aim	Tool	Running platform	Input	Ref
Modeling HLA-B*57:01-peptide structures	TFold	deployed locally via python	MHC allele and peptide sequence	[9]
Docking abacavir to the HLA-B*57:01-peptide structures	AutoDock Vina	deployed locally via python	HLA-B*57:01-peptide structure and SMILES of abacavir	[10, 11]
Modeling HLA-B*57:01-compound structures	Chai	deployed locally via python	sequence of HLA-B*57:01 and SMILES of candidate compound	[1]
	Boltz-2	https://www.tamarind.bio/	sequence of HLA-B*57:01 and SMILES of candidate compound	[2]
	diffDock	https://www.tamarind.bio/	structural data file of both HLA-B*57:01 and candidate compound	[3]
	AutoDock Vina	deployed locally via python	HLA-B*57:01 structure and SMILES of candidate compound	[10, 11]
Quantifying binding energy of Chai modeled HLA-B*57:01-compound structures	AutoDock Vina	deployed locally via python	HLA-B*57:01-compound structure	[10, 11]
Docking peptides to the HLA-B*57:01-compound structures	AutoDock CrankPep	deployed locally via python	structural data files of both HLA-B*57:01-compound complex and candidate peptide	[4]
	HADDOCK	https://rascar.science.uu.nl/haddock2.4/	structural data files of both HLA-B*57:01-compound complex and candidate peptide	[5]
	HPEPDOCK	http://huanglab.phys.hust.edu.cn/hpepdock/	structural data files of both HLA-B*57:01-compound complex and candidate peptide	[6]
	CABS-dock	https://biocomp.chem.uw.edu.pl/CABSdock/	HLA-B*57:01-compound structure and peptide sequence	[7]
	GalaxyPepDock	https://galaxy.seoklab.org/cgi-bin/submit.cgi?type=PEPDOCK	HLA-B*57:01-compound structure and peptide sequence	[8]

Table S2. Benchmarking TFold using unseen Crystallized HLA-peptide structures.

PDB ID	MHC allele	Peptide squence	Peptide length	Overall RMSD (in Å)	Binding groove RMSD (in Å)
8tub	HLA-B*07:02	HPNGYKSLSTL	11	0.32	0.31
8tuh	HLA-B*07:02	RPIIRPATL	9	0.35	0.35
8rjh	HLA-A*24:02	NYNYLFRLF	9	0.42	0.36
8rji	HLA-A*24:02	NYNYRYRLF	9	0.42	0.36
8rro	HLA-A*03:01	VVVGAVGVGK	10	0.38	0.42
8xg2	HLA-A*26:01	EVFNATRFASVY	12	0.44	0.43
8xkc	HLA-A*26:01	CVADYSVLY	9	0.38	0.37
8f7m	HLA-B*57:01	TSNLQEQIGW	10	0.24	0.23
8xes	HLA-A*26:01	DVMTYIHSM	9	0.39	0.41
8xfz	HLA-A*26:01	YYVARTNIYY	9	0.40	0.41
8rni	HLA-A*03:01	VVVGAVGVGK	10	0.38	0.42
8vjz	HLA-A*03:01	VVVGAGGVGK	10	0.37	0.41
8xke	HLA-A*26:01	EVDNATRFASVY	12	0.44	0.45
8fu4	HLA-A*02:01	TLFDEPPPL	9	0.45	0.45
8v4z	HLA-B*35:01	LPFEKSTIM	9	0.39	0.41
8v50	HLA-B*35:01	LPFDKSTVM	9	0.39	0.41
8v51	HLA-B*35:01	LPFEKSTVM	9	0.36	0.36
8f5a	HLA-B*57:01	TSTLQEQIGW	10	0.24	0.24
8rj5	HLA-A*24:02	NYNYLYRLF	9	0.38	0.36
8rne	HLA-E*01:03	VMPLSAPTL	9	0.49	0.45
8tmu	HLA-B*73:01	NRFAGFGIGL	10	0.35	0.33
8tq6	HLA-B*44:05	EEFGRAFSF	9	0.29	0.30
9bl2	HLA-B*57:03	ASLNLPAVSW	10	0.26	0.25
9bl3	HLA-B*57:03	ASLNLPAVSW	10	0.26	0.25
9bl4	HLA-B*57:03	ASLNLPAVSW	10	0.26	0.25
9bl5	HLA-A*24:02	TYQWIIRNW	9	0.38	0.39
9bl6	HLA-A*24:02	TYQWIIRNW	9	0.38	0.39
9bl9	HLA-A*24:02	RYPLTFGW	8	0.38	0.37
9bla	HLA-A*24:02	RYPLTFGW	8	0.38	0.37
8emj	HLA-B*35:01	LPFDIATIM	9	0.40	0.41
8emk	HLA-B*35:01	LPFDKPTIM	9	0.39	0.40
8k4t	HLA-A*11:01	VVVGACGVGK	10	0.37	0.39
8k4v	HLA-A*11:01	VVVGARGVGK	10	0.43	0.42
8k50	HLA-A*11:01	VVVGARGVGK	10	0.43	0.42
8rnf	HLA-E*01:03	VIPLSAPTL	9	0.48	0.44
8tq5	HLA-B*44:05	EEFGRAFSF	9	0.29	0.30
8vcl	HLA-A*03:01	ALHGGWTTK	9	0.36	0.44
8emf	HLA-B*35:01	LPFDKSTVM	9	0.39	0.41
8emg	HLA-B*35:01	LPFEKSTIM	9	0.39	0.41

8emi	HLA-B*35:01	LPFDKATIM	9	0.40	0.41
8rym	HLA-A*03:01	ELFSYLIEK	9	0.40	0.42
8ryn	HLA-A*11:01	ELFSYLIEK	9	0.38	0.47
8ryp	HLA-A*03:01	ELFSYLIEK	9	0.40	0.42
8u9g	HLA-A*02:01	KLSHQLVLL	9	0.45	0.46
8en8	HLA-B*35:01	LPFDKSTIM	9	0.37	0.36
8rlt	HLA-E*01:03	ILSPFLPLL	9	0.52	0.42
8rlu	HLA-E*01:03	ILNPFLPLL	9	0.51	0.41
8rlv	HLA-E*01:03	ILSPFIPLL	9	0.51	0.42
8ryo	HLA-A*03:01	ELFSYLIEK	9	0.40	0.42
8ryq	HLA-A*11:01	ELFSYLIEK	9	0.38	0.47
8tbv	HLA-A*02:01	KLSHQLVLL	9	0.45	0.46
8tbw	HLA-A*02:01	KLSHQPVLL	9	0.43	0.42
8enh	HLA-B*35:01	LPFEKSTIM	9	0.39	0.41
8eo8	HLA-B*35:01	LPFDKATIM	9	0.40	0.41
8rbv	HLA-A*02:01	VLNDILARL	9	0.43	0.47
8wte	HLA-A*11:01	VVGAVGVGK	9	0.40	0.41
9dl1	HLA-A*02:01	SLLMWITQV	9	0.41	0.42
8rng	HLA-B*18:01	TEVETYVL	8	0.28	0.30
8rnh	HLA-B*18:01	EEIEITTHF	9	0.37	0.37
8roo	HLA-B*18:01	YERMCNIL	8	0.27	0.29
8rop	HLA-B*18:01	QEIRTFSF	8	0.33	0.36
8ye4	HLA-A*24:02	NYNYLYRLF	9	0.38	0.36
9asg	HLA-A*03:01	ALHGGWTTK	9	0.36	0.44
8wul	HLA-A*11:01	VVGAVGVGK	9	0.40	0.41
9asf	HLA-A*03:01	AHHGGWTTK	9	0.37	0.44
8rbu	HLA-A*11:01	SVLNDILARL	10	0.40	0.47
8rcv	HLA-B*13:01	SVLNDIFSRL	10	0.27	0.26
8ref	HLA-B*13:01	SVLNDILARL	10	0.28	0.27
8rh6	HLA-A*11:01	SVLNDILSRL	10	0.40	0.43
8rhq	HLA-A*11:01	SVLNDIFSRL	10	0.40	0.47
9c6x	HLA-B*39:01	NRVMLPKAA	9	0.33	0.28
9c6v	HLA-B*39:06	NRVMLPKAA	9	0.34	0.30
9c6w	HLA-B*39:06	NRVMLPKAA	9	0.34	0.30

Table S3. Centroid distances (in Å) between redocked abacavir poses and native pose.

	AutoDock Vina	diffDock	Boltz-2	Chai
Redocked abacavir vs 3upr	3.4	11.3	0.5	0.3
Redocked abacavir vs 3vri	3.5	11.3	0.6	0.5
Redocked abacavir vs 3vrj	3.4	11.4	0.7	0.6
Redocked abacavir vs 5u98	3.3	11.4	0.6	0.4

Table S4. Comparison of quantitative differences in peptide repertoire upon abacavir binding.

Key amino acid		Number of peptides	Average contact number difference of the whole complex*		Average contact number difference at anchor points*		Average ADCP binding energy difference	
			Value	P value	Value	P value	Value	P value
P1_K	Yes	2845	-4.26	8.72E-06	-7.41	4.10E-11	4.67	8.27E-13
	No	10861	-9.60		-12.32		4.98	
P4_P	Yes	1794	-12.95	3.88E-04	-11.62	6.80E-01	4.91	9.10E-01
	No	11912	-7.82		-11.25		4.92	
P4_K	Yes	1224	-1.12	2.15E-06	-7.00	8.17E-06	4.99	2.14E-01
	No	12482	-9.22		-11.72		4.91	
P9_W	Yes	7308	-27.70	<0.00E-20	-26.88	<0.00E-20	5.52	<0.00E-20
	No	6398	13.44		6.49		4.24	

* Calculated using MDAnalysis

Table S5. Selected peptides (n=366) containing amino acids at P1, P4 and P9 that only favor positive abacavir analogues.

Peptide sequence	Peptide lenght	Category
LTLPSHSLEHL	11	P5-x
LTDPNRPRFTL	11	P5-x
LSRPERPDLVF	11	P5-x
LSSASLSQVFL	11	P5-x
LTSPEELYRVF	11	P5-x
LTTAVLAERLF	11	P5-x
MSLPSTPDIKI	11	P5-x
MTNPSRAGVIL	11	P5-x
MSFPVGDVNNF	11	P5-x
LVNPKQQEKLF	11	P5-x
MAMPADTNKAF	11	P5-x
LSATIPNARQF	11	P5-x
IGSTIDDTISKF	12	P5-x
ISFPATGCQKLI	12	P5-x
ISSPSLSEKQYF	12	P5-x
ATLPNYEDVATF	12	P5-x
GLALAAFLGLVL	12	Other
ITYPYTPQFKVF	12	P5-x
ILMPVNKVVQSF	12	P5-x
ISKPVASDSTYF	12	P5-x
AVCPYCSLRFF	11	P5-x
ASNPYSGDLTKF	12	P5-x
LVDPNHSGLVTF	12	P5-x
QASQAVSKQQVF	12	Other
MSFPTGKSDRGF	12	P5-x
LSLPAEFPDKVF	12	P5-x
LSKPVSSYPNTF	12	P5-x
INVTPIAALLYT	12	P1x
LVPPISKPPPGF	12	P-x
GRGTSTYDGFGLAW	14	Other
ITKPGSIDSNNQLF	14	P5-x
ITFPMIGTGSLQF	13	P5-x
LAAPFTDQIFHF	12	P5-x
LSRPSLLITDSF	12	P5-x
LSIPWGIKQAGF	12	P5-x
LSIPYRLIFEKF	12	P5-x

LSLPDLRCSLLL	12	P5-x
LSMAMQKIYQTF	12	P5-x
MTFPTGYIFDRF	12	P5-x
LTVPVETVLRVL	12	P5-x
ITYPAGFMDVI	11	P5-x
IVFPVPGICQF	11	P5-x
ITCPADPKKTL	11	P5-x
ITFPYGYHAGF	11	P5-x
ITKPASVFVQL	11	P5-x
IGKPAHLIVSL	11	P5-x
IGMPEPDAQRF	11	P5-x
ISFPATGCQKL	11	P5-x
ISNPEFAFLRF	11	P5-x
ISLPTSKNWTF	11	P5-x
ISLPINLKTVF	11	P5-x
ISFPEKELLLL	11	P5-x
ISGPQQTQKVF	11	P5-x
ISIANGLINEI	11	P5-x
LASTLKTLLFF	11	P5-x
LAVPDNYKVIF	11	P5-x
LASPDLGLLKL	11	P5-x
LCKPETVPVDF	11	P5-x
LARPAALTALL	11	P5-x
ATAALLGHILL	11	P5-x
ALSAVVSLLQF	11	P5-x
ASAPSILIHFI	11	P5-x
ASCPNHFEGLF	11	P5-x
ATSTIKALLEL	11	P5-x
ATIPVSQISTI	11	P5-x
AAVPQDVVRQF	11	P5-x
ACRAIGILSRF	11	P5-x
ISKPVGFGEEF	11	P5-x
GTYTDVTPRQF	11	P5-x
GYSALRAQETF	11	P5-x
IAFAPNLGRSF	11	P5-x
IALPAQGLIEF	11	P5-x
DLTTPYRIFSF	11	Other
GALAPPAPIKF	11	P5-x
MTLPSIGIPLL	11	P5-x
MTVPSKLWGFF	11	P5-x
MTFPAGIVRVF	11	P5-x

MGNPQRALLYL	11	P5-x
MSFPSGIIKVF	11	P5-x
LSIPDEQLHSF	11	P5-x
LSKTAETDVLF	11	P5-x
LSLPDLRCSLL	11	P5-x
LSLPDTWMKEF	11	P5-x
LSHPLGIVQGF	11	P5-x
LSATVKIIQML	11	P5-x
LSFPESPSIEF	11	P5-x
LSLPEDVLFHI	11	P5-x
LSYPDCKLQTL	11	P5-x
LTDPSKTSNTI	11	P5-x
LSSTEKSQIEF	11	P5-x
LSRPDGSASFL	11	P5-x
LSSAHLAFMTF	11	P5-x
LSSPEQIQKQL	11	P5-x
LSLPSSWDYRF	11	P5-x
ATCPHWVF	8	P1-P9
ASRPPVQI	8	P1-P9
GTRPPHVF	8	P1-P9
GTGASGSF	8	P1-P9
ASRPPVTL	8	P1-P9
ISKPPPGF	8	P1-P9
LARPSSTITYL	11	P5-x
ASLPAELINQI	11	P5-x
ASLPSELIVHI	11	P5-x
ASFARVGGRLF	11	P5-x
ATMPHQLQDTF	11	P5-x
ATNALLNSLEF	11	P5-x
ATVAAELLNHL	11	P5-x
ISAPDKRIYQF	11	P5-x
ISDPYGNLKDF	11	P5-x
ISKPTEWSDEL	11	P5-x
ISLPDDVRRRL	11	P5-x
ILDPVHSVRLF	11	P5-x
IAIPDASKLQF	11	P5-x
AVWATSRSF	9	P1-P9
ATLALRTKF	9	P1-P9
ATLAEIHHF	9	P1-P9
ATKTFVDFF	9	P1-P9
ATKAVLQEF	9	P1-P9
ATDTKNVQF	9	P1-P9

ATNPISRVL	9	P1-P9
ATNPESKVF	9	P1-P9
GVHTVHVTF	9	P1-P9
GSNPVRVSF	9	P1-P9
GALAVLQQF	9	P1-P9
GSRPSDARF	9	P1-P9
GTMPLRNIF	9	P1-P9
AAVTIQKAF	9	P1-P9
ASYPELRLF	9	P1-P9
ASNPRVTRF	9	P1-P9
ASLPLRVSF	9	P1-P9
ASLAIKPVF	9	P1-P9
AATTVLRQL	9	P1-P9
AANPHSFVF	9	P1-P9
AALAVLKGF	9	P1-P9
AAGPIRVVL	9	P1-P9
LSKTSVVDL	9	P1-P9
LSLPRRGSF	9	P1-P9
AILTNPVTF	9	P1-P9
ASLPQAATF	9	P1-P9
ITGPIAKLI	9	P1-P9
ITQPTLHSF	9	P1-P9
ITTTINPRF	9	P1-P9
GHKPESVDL	9	P1-P9
GHRTGTLEF	9	P1-P9
AAATRTTSF	9	P1-P9
AAKPHNPGF	9	P1-P9
AARTIQTAF	9	P1-P9
ITKTGAKLL	9	P1-P9
ASNTHIFTF	9	P1-P9
ATHPSRAQF	9	P1-P9
ATIPNARQF	9	P1-P9
GTIAIKHYF	9	P1-P9
GTIPKRHEL	9	P1-P9
LTRPPNYF	8	P1-P9
LSGPRLLF	8	P1-P9
GSNPHLQTF	9	P1-P9
GTATSQRFF	9	P1-P9
GTTTLAFKF	9	P1-P9
ISSTVQRQL	9	P1-P9
ISAAKDVKL	9	P1-P9
LSRPEAQSL	9	P1-P9

LSSPVTKSF	9	P1-P9
LINPQGKAF	9	P1-P9
LGAPAKPPL	9	P1-P9
MAWTKYQLF	9	P1-P9
MTKTYIPIL	9	P1-P9
MSLPYRLVF	9	P1-P9
MSLPKLESF	9	P1-P9
MSKAPSNTF	9	P1-P9
LVAPVPHSF	9	P1-P9
LTHPFALTL	9	P1-P9
LTDPNRPRF	9	P1-P9
LSYPKNESF	9	P1-P9
LSVPRDWKF	9	P1-P9
LSVPNLPEI	9	P1-P9
LTKPFEYLF	9	P1-P9
LTRPNIYLI	9	P1-P9
LTRPGSSYF	9	P1-P9
LTLPVNHIF	9	P1-P9
LTKTHHDMF	9	P1-P9
LTRTELETL	9	P1-P9
IALPSLRIL	9	P1-P9
ISNPVTKEMLF	11	P5-x
ITSPSTPSKKF	11	P5-x
ITNADSATRLL	11	P5-x
IVFPEDGIHGF	11	P5-x
ISRPEAAQQAF	11	P5-x
ISVPHKIITHL	11	P5-x
ISYPQEVIPTF	11	P5-x
ISFPLKEYF	9	P1-P9
ISFPDPKML	9	P1-P9
ISAPLVKTL	9	P1-P9
IQRPNSFLF	9	P1-P9
ISKAHQEEI	9	P1-P9
IVKPNIAHF	9	P1-P9
ITYPYTPQF	9	P1-P9
ITSPVHVSF	9	P1-P9
ITKTVVENI	9	P1-P9
ISRPVPVRF	9	P1-P9
ISRPIVNLF	9	P1-P9
ISRAVVLVF	9	P1-P9
ISLAAQKFI	9	P1-P9
ISKTLQRTL	9	P1-P9

ISKPDVITL	9	P1-P9
ITKTQKVRF	9	P1-P9
ITFTKNNQF	9	P1-P9
ITFPGIKLI	9	P1-P9
ITFAPTYRF	9	P1-P9
ISVPIFKQF	9	P1-P9
ISTPVIRTF	9	P1-P9
LSKPNPPSL	9	P1-P9
LSHAITLEF	9	P1-P9
LSFTHPTSF	9	P1-P9
LSFTHPISF	9	P1-P9
LSAPPHFHF	9	P1-P9
LSSPFLKGF	9	P1-P9
LSRPPTEQL	9	P1-P9
LSRPNVLSF	9	P1-P9
LSKTEFLSF	9	P1-P9
LARALSPAF	9	P1-P9
LAFAGRYAF	9	P1-P9
LIQAKVGSF	9	P1-P9
LAYPNGHYF	9	P1-P9
LSYPDNFLHI	10	P5-x
LSRPTTETQF	10	P5-x
LSIPDIKTAF	10	P5-x
LSKPVDVSKF	10	P5-x
LSKPVPESEF	10	P5-x
LSKTETNVYF	10	P5-x
LSLPEDFYHF	10	P5-x
LSNAYAREEF	10	P5-x
LSRPDGSASF	10	P5-x
MSLPTPLPGF	10	P5-x
LVYPGDPLRF	10	P5-x
MAGPLRAPLL	10	P5-x
ASMADKRIHF	10	P5-x
ATFPLSVQKF	10	P5-x
ATIAEIKNLF	10	P5-x
ASLPTHLITF	10	P5-x
ISNPKTAEF	9	P1-P9
IVKPLNPNF	9	P1-P9
IWQAYLDYL	9	P1-P9
ISSPWGKHVF	10	P5-x
ISSTAFDIRF	10	P5-x
ISVAALSKIF	10	P5-x

ISAAKTGVRF	10	P5-x
ISHPDVGRYF	10	P5-x
ISIPAVQKVF	10	P5-x
SLADAINTEF	10	P-x
SLLVTLVVLF	10	P-x
AIRTGSPLSL	10	P5-x
DIERPTYTNL	10	P-x
ASIPTTVGSL	10	P5-x
ISQPASGNTF	10	P5-x
ITRAVLEQFL	10	P5-x
SGSSATRSLF	10	P-x
MTRPDLPVGF	10	P5-x
MVEPENAVTI	10	P5-x
ISLPNGLQGF	10	P5-x
LTIPDGGVHI	10	P5-x
LTMPDTPRLF	10	P5-x
ISKPGQFETF	10	P5-x
ISDPYKVYRI	10	P5-x
AAAAANPQTL	10	P-x
GSHPWHWYF	9	P1-P9
ISAALLMACSL	11	P5-x
GAVAMFPPTSI	11	P5-x
ASYPHCLQF	9	P1-P9
ATKTVIYLL	9	P1-P9
AMYPHIFYF	9	P1-P9
ASKTVFFFF	9	P1-P9
ASLAAVHLF	9	P1-P9
ASLPALLLI	9	P1-P9
ASLPFFEVF	9	P1-P9
AVNPLLRVI	9	P1-P9
ISRPNPPNF	9	P1-P9
ISSPVVTSL	9	P1-P9
ISVANNRLF	9	P1-P9
ITAALRDLF	9	P1-P9
ISIAVIHHF	9	P1-P9
ISLPFSTAF	9	P1-P9
ISLPIHPMI	9	P1-P9
ISNPAPQQF	9	P1-P9
ITSPTSEEF	9	P1-P9
ITVPIGLYF	9	P1-P9
ITVPVFHLF	9	P1-P9
ITYPMLFKL	9	P1-P9

ITNPKLDFF	9	P1-P9
ITCPKVNQF	9	P1-P9
ITDTYKLLF	9	P1-P9
ITFPIIVHF	9	P1-P9
ITFPIQKVL	9	P1-P9
ITKPFFAAI	9	P1-P9
ITMTVAHEL	9	P1-P9
IAMPLHMIF	9	P1-P9
GTSPLLWYF	9	P1-P9
LSNTSQKSL	9	P1-P9
LTKPRDSTI	9	P1-P9
LSNTVMPRF	9	P1-P9
LTSTLQVFF	9	P1-P9
ISIPIGFLF	9	P1-P9
LAHAIRLLL	9	P1-P9
LHRTTNFFI	9	P1-P9
LASPIYTHF	9	P1-P9
LAVPFCVNF	9	P1-P9
LVLPIAYEF	9	P1-P9
MAIPLVFQI	9	P1-P9
LTRTLQVLL	9	P1-P9
LTVPFARYL	9	P1-P9
MSQALKATF	9	P1-P9
LSIPYRLIF	9	P1-P9
LSKPNIFIL	9	P1-P9
LSLPGILHF	9	P1-P9
LSAALHLLL	9	P1-P9
LSFPNLFKF	9	P1-P9
LTFAGLCYF	9	P1-P9
LTLPLARLL	9	P1-P9
LSRPNIFIL	9	P1-P9
LSRTLLAFF	9	P1-P9
LSTTIQEEF	9	P1-P9
LSTTVQPNL	9	P1-P9
LSVALLRHF	9	P1-P9
LSYPQTDVF	9	P1-P9
LSWPSPWVI	9	P1-P9
LSVAVRSEF	9	P1-P9
IVYPGIAVFF	10	P5-x
ISNTASYLRL	10	P5-x
ISQPSPQASF	10	P5-x
ISRTPVLMNF	10	P5-x

ISMPDVDLHL	10	P5-x
ISKPSVSAFF	10	P5-x
ISLADIAQKL	10	P5-x
ISMPDFDLHL	10	P5-x
ISWTGDGWEF	10	P5-x
ITKASVSITF	10	P5-x
ITKPPDGSAF	10	P5-x
ITLANVIRQL	10	P5-x
ITLPEEFHDF	10	P5-x
ITFPIQKVLF	10	P5-x
LCWAEGQRLF	10	P5-x
LGPPELLFLL	10	P5-x
LAQAENLQAL	10	P5-x
LSLAPDTRLF	10	P5-x
LSLPDIKVYL	10	P5-x
LSLPDLRCSL	10	P5-x
LSAAILDKNF	10	P5-x
LSAANIQPIF	10	P5-x
LSLPESTESF	10	P5-x
ATLAAVLQRI	10	P5-x
ATLTIQGIRF	10	P5-x
ATWADIFKRF	10	P5-x
AVKAELRQYF	10	P5-x
MFKTLHGSALF	11	P5-x
ASAPRLLLLF	10	P5-x
ASIAAGVLFF	10	P5-x
GSGPIQLWQF	10	P5-x
GSLTAESQEL	10	P5-x
LSLPQEPYSF	10	P5-x
MTVPSKLWGF	10	P5-x
MLKPHCTVSF	10	P5-x
LVRPHIDISF	10	P5-x
LSNPDPRLLF	10	P5-x
LTVTDFKLYF	10	P5-x
LTLADLRVLF	10	P5-x
LTLPDLAEQF	10	P5-x
LSDTVASDPGVL	12	P5-x

Table S6. Prediction performances and corresponding hyperparameters of support vector machine models using different training sets.

Feature	sigma	C	AUC	Sensitivity	Specificity	Accuracy
Selected peptides (n=366), SVM	1.55E-03	0.25	1	0.875	1	0.933
4+null	2.69E-01	1	0.946	0.875	0.714	0.8
3upr	2.11E+01	0.5	0.804	1	0.714	0.867
3vri	8.49E-01	4	0.661	0.375	0.571	0.467
5u98	1.17E+01	0.25	0.625	0.75	0.714	0.733
3vrj	3.54E-01	4	0.411	0.375	0.571	0.467
null	2.97E+01	1	0.411	0.5	0.429	0.467

References

1. Discovery C, Boitreaud J, Dent J et al. Chai-1: Decoding the molecular interactions of life, bioRxiv 2024:2024.2010.2010.615955.
2. Passaro S, Corso G, Wohlwend J et al. Boltz-2: Towards Accurate and Efficient Binding Affinity Prediction, bioRxiv 2025:2025.2006.2014.659707.
3. Corso G, Stärk H, Jing B et al. DiffDock: Diffusion Steps, Twists, and Turns for Molecular Docking. 2022, arXiv:2210.01776.
4. Zhang Y, Sanner MF. AutoDock CrankPep: combining folding and docking to predict protein-peptide complexes, Bioinformatics 2019;35:5121-5127.
5. Dominguez C, Boelens R, Bonvin AM. HADDOCK: a protein-protein docking approach based on biochemical or biophysical information, J Am Chem Soc 2003;125:1731-1737.
6. Zhou P, Jin B, Li H et al. HPEPDOCK: a web server for blind peptide-protein docking based on a hierarchical algorithm, Nucleic Acids Res 2018;46:W443-w450.
7. Kurcinski M, Jamroz M, Blaszczyk M et al. CABS-dock web server for the flexible docking of peptides to proteins without prior knowledge of the binding site, Nucleic Acids Res 2015;43:W419-424.
8. Lee H, Heo L, Lee MS et al. GalaxyPepDock: a protein-peptide docking tool based on interaction similarity and energy optimization, Nucleic Acids Res 2015;43:W431-435.
9. Mikhaylov V, Brambley CA, Keller GLJ et al. Accurate modeling of peptide-MHC structures with AlphaFold, Structure 2024;32:228-241.e224.
10. Trott O, Olson AJ. AutoDock Vina: improving the speed and accuracy of docking with a new scoring function, efficient optimization, and multithreading, J Comput Chem 2010;31:455-461.
11. Eberhardt J, Santos-Martins D, Tillack AF et al. AutoDock Vina 1.2.0: New Docking Methods, Expanded Force Field, and Python Bindings, J Chem Inf Model 2021;61:3891-3898.
