# Supplementary material for: Prediction of drug hypersensitivity by comprehensive modeling of HLA-peptidomes
Source: Brief Bioinform. 2026 Jul 3;27(4):bbag350. doi: 10.1093/bib/bbag350 (PMC13331351; doi:10.1093/bib/bbag350)
Supplement: Supplementary_Figure_legends_bbag350 [file supplementary_figure_legends_bbag350.docx]

**Supplementary Figure legends**

**Supplementary Figure 1. Utilization of molecular docking and structural modeling tools.** (A) TFold (modeling HLA-peptide structures) and Autodock Vina (molecule docking) were used to assess conventional approach in studying HLA-compound interactions. (B) Other molecular docking and structural modeling tools were evaluated in order to establish a novel pipeline in modeling HLA-B*57:01-compound-peptide complexes. (C) Autodock Vina was used to derive binding energy from Chai-modeled HLA-B*57:01-compound structures.

**Supplementary Figure 2. Binding energy distribution of the HLA-B*57:01-presented repertoire from ADCP, categorized by peptide length.**

**Supplementary Figure 3. Chai-modeled flucloxacillin binding pose in comparison with abacavir within the HLA-B*57:01 binding cleft.**
